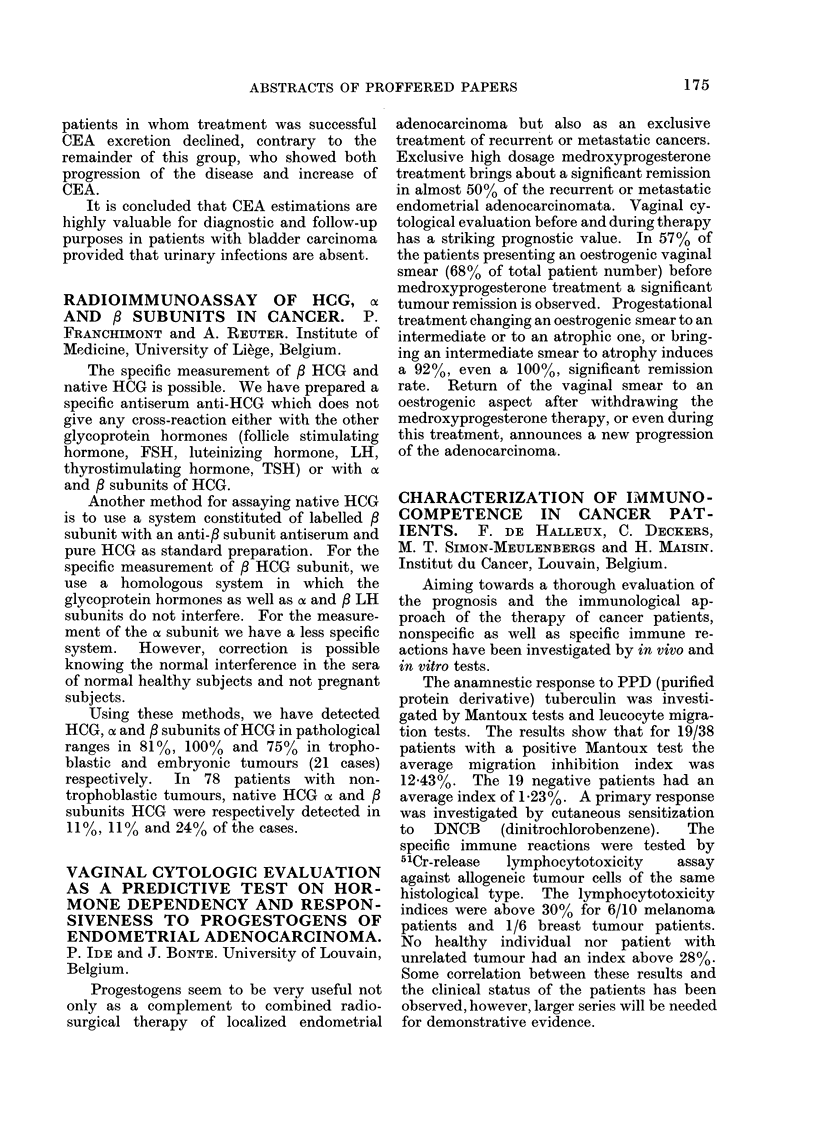# Proceedings: Radioimmunoassay of HCG, alpha and beta subunits in cancer.

**DOI:** 10.1038/bjc.1974.138

**Published:** 1974-08

**Authors:** P. Franchimont, A. Reuter


### RADIOIMMUNOASSAY OF HCG, $\alpha$ AND $\beta$ SUBUNITS IN CANCER.

P. FRANCHIMONT and A. REUTER. Institute of Medicine, University of Liège, Belgium.

The specific measurement of $\beta$ HCG and native HCG is possible. We have prepared a specific antiserum anti-HCG which does not give any cross-reaction either with the other glycoprotein hormones (follicle stimulating hormone, FSH, luteinizing hormone, LH, thyrostimulating hormone, TSH) or with $\alpha$ and $\beta$ subunits of HCG.

Another method for assaying native HCG is to use a system constituted of labelled $\beta$ subunit with an anti-$\beta$ subunit antiserum and pure HCG as standard preparation. For the specific measurement of $\beta$ HCG subunit, we use a homologous system in which the glycoprotein hormones as well as $\alpha$ and $\beta$ LH subunits do not interfere. For the measurement of the $\alpha$ subunit we have a less specific system. However, correction is possible knowing the normal interference in the sera of normal healthy subjects and not pregnant subjects.

Using these methods, we have detected HCG, $\alpha$ and $\beta$ subunits of HCG in pathological ranges in 81%, 100% and 75% in trophoblastic and embryonic tumours (21 cases) respectively. In 78 patients with non-trophoblastic tumours, native HCG $\alpha$ and $\beta$ subunits HCG were respectively detected in 11%, 11% and 24% of the cases.